# The role of Chinese herbal medicine in the treatment of diabetic nephropathy by regulating endoplasmic reticulum stress

**DOI:** 10.3389/fphar.2023.1174415

**Published:** 2023-06-26

**Authors:** Maoying Wei, Xingxing Liu, Mingdi Li, Xiaochan Tian, Mingyue Feng, Boxian Pang, Zeyang Fang, Junping Wei

**Affiliations:** ^1^ Department of Endocrinology, Guang’Anmen Hospital, China Academy of Chinese Medical Sciences, Beijing, China; ^2^ Department of Emergency, Guang’Anmen Hospital, China Academy of Chinese Medical Sciences, Beijing, China

**Keywords:** Chinese herbal medicine, diabetic nephropathy, endoplasmic reticulum stress, mechanism, renal protection

## Abstract

Diabetic nephropathy (DN), a prevalent microvascular complication of diabetes mellitus, is the primary contributor to end-stage renal disease in developed countries. Existing clinical interventions for DN encompass lifestyle modifications, blood glucose regulation, blood pressure reduction, lipid management, and avoidance of nephrotoxic medications. Despite these measures, a significant number of patients progress to end-stage renal disease, underscoring the need for additional therapeutic strategies. The endoplasmic reticulum (ER) stress response, a cellular defense mechanism in eukaryotic cells, has been implicated in DN pathogenesis. Moderate ER stress can enhance cell survival, whereas severe or prolonged ER stress may trigger apoptosis. As such, the role of ER stress in DN presents a potential avenue for therapeutic modulation. Chinese herbal medicine, a staple in Chinese healthcare, has emerged as a promising intervention for DN. Existing research suggests that some herbal remedies may confer renoprotective benefits through the modulation of ER stress. This review explores the involvement of ER stress in the pathogenesis of DN and the advancements in Chinese herbal medicine for ER stress regulation, aiming to inspire new clinical strategies for the prevention and management of DN.

## 1 Introduction

Diabetes mellitus (DM) constitutes an escalating global public health concern. As reported by the International Diabetes Federation, the prevalence of DM surged to 536.6 million individuals globally in 2021, with projections indicating a rise to 783.2 million by 2045 (Sun et al., 2022). A significant proportion, ranging from 20% to 40% of these individuals, concurrently live with diabetic kidney disease (de Boer et al., 2011; Afkarian et al., 2016). Diabetic nephropathy (DN), a form of renal damage precipitated by chronic hyperglycemia, can affect the entire kidney structure, including the glomerulus, renal tubules, renal interstitium, and renal vessels. Clinically, DN is characterized by persistent albuminuria and/or a progressive decrease in glomerular filtration rate, which can eventually progress to end-stage renal disease. Given the complex pathogenesis of DN, no curative treatment has been established to date. Existing therapeutic regimens primarily focus on glycemic control, blood pressure management, cardiovascular risk reduction, and inhibition of the renin-angiotensin system (Umanath and Lewis, 2018). In recent years, novel hypoglycemic agents, namely, sodium-glucose cotransporter 2 inhibitors and glucagon-like peptide 1 receptor agonists, have garnered significant attention for their renoprotective effects (Alicic et al., 2019; Kristensen et al., 2019). Despite these efforts, one-third of patients ultimately progress to end-stage renal disease necessitating renal replacement therapy (Collins et al., 2012). Therefore, there is currently an urgent need to identify novel therapeutic strategies that can impede the progression of DN.

Recent advancements in research have unveiled the potential significance of endoplasmic reticulum (ER) stress in the pathogenesis and progression of DN. Elevations in ER stress markers, namely, glucose-regulated protein 78 (GRP78) and the C/EBP homologous protein (CHOP), have been observed in the renal tissue of DN patients. These molecular alterations are often associated with histopathological aberrations such as glomerulosclerosis, tubular atrophy, and interstitial fibrosis (Pang et al., 2016). Notably, several DN-associated factors, including proteinuria, hyperglycemia, free fatty acids, and advanced glycation end products (AGEs), have been reported to trigger ER stress, thereby contributing to renal intrinsic cell damage (Lindenmeyer et al., 2008; Park et al., 2017; Jeong and Lee, 2021). Recent studies have underscored the renoprotective effects of ER stress inhibition, thereby highlighting its therapeutic potential (Yuan et al., 2018; Xiong et al., 2020). In this context, the strategy of normalizing ER stress through pharmacological interventions has been postulated as an effective approach to curtail DN progression (Chen H. Y. et al., 2019). Moreover, an increasing body of evidence suggests the potential of Chinese herbal medicine in attenuating DM-induced renal damage by modulating ER stress. This review article explores the critical role of ER stress in the pathogenesis of DN and the pertinent advancements in the field of Chinese herbal medicine for the prevention and treatment of DN via ER stress regulation.

## 2 ER stress and the unfolded protein response

**FIGURE 1:**
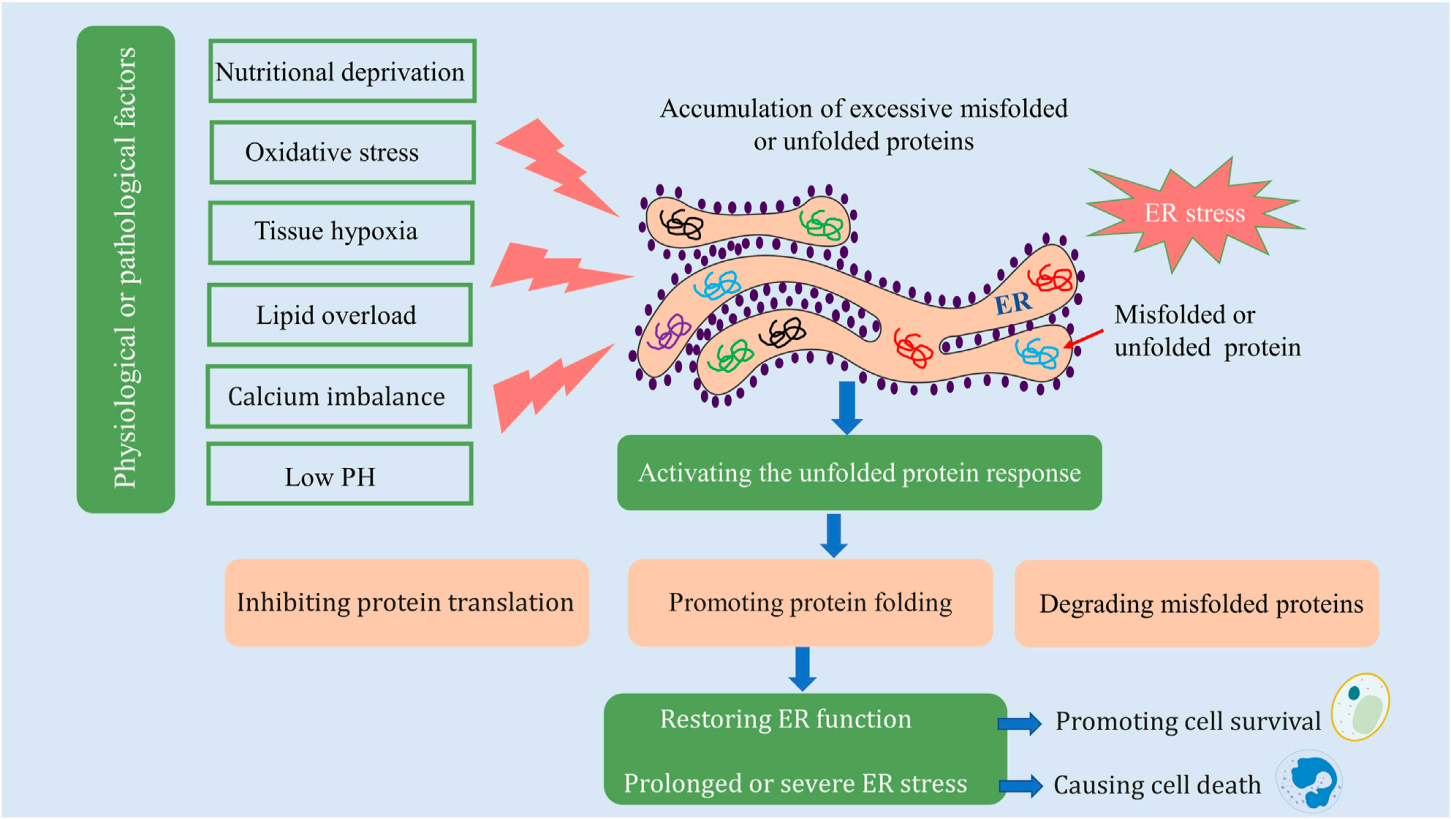
ER stress and the unfolded protein response. Materials provided by Servier Medical Art (smart.servier.com).

The ER is an intracellular organelle found in eukaryotic cells, critical for various functions including protein synthesis and folding, lipid biosynthesis, calcium storage, and detoxification processes (Schwarz and Blower, 2016; Huang et al., 2022). The ER is highly sensitive to environmental alterations, and several physiological or pathological conditions can adversely affect its function. Factors such as nutritional deprivation, oxidative stress, tissue hypoxia, lipid overload, calcium imbalance, and low pH can precipitate the accumulation of unfolded or misfolded proteins within the ER, thereby impairing its function and precipitating what is known as ER stress. The manifestation of ER stress can be categorized into three primary responses: the unfolded protein response (UPR), the ER overload response, and the sterol regulatory element-binding protein response. The first two responses arise from disturbances in protein processing, whereas the latter response is a consequence of cholesterol depletion synthesized at the endoplasmic reticulum’s surface. The UPR, the most extensively studied of these responses, plays a pivotal role in mitigating damage induced by ER stress. In response to ER stress, cells initiate the UPR to alleviate damage, including inhibition of protein translation, enhancement of protein folding capabilities, and degradation of misfolded proteins via ER-associated degradation (Engin and Hotamisligil, 2010). This response mechanism assists in restoring ER homeostasis and promotes cell survival. However, in cases where ER stress is either too severe or sustained for extended periods, the UPR may paradoxically activate programmed cell death (Engin and Hotamisligil, 2010; So, 2018) (Figure 1).

## 3 Signaling pathways for the unfolded protein response

**FIGURE 2:**
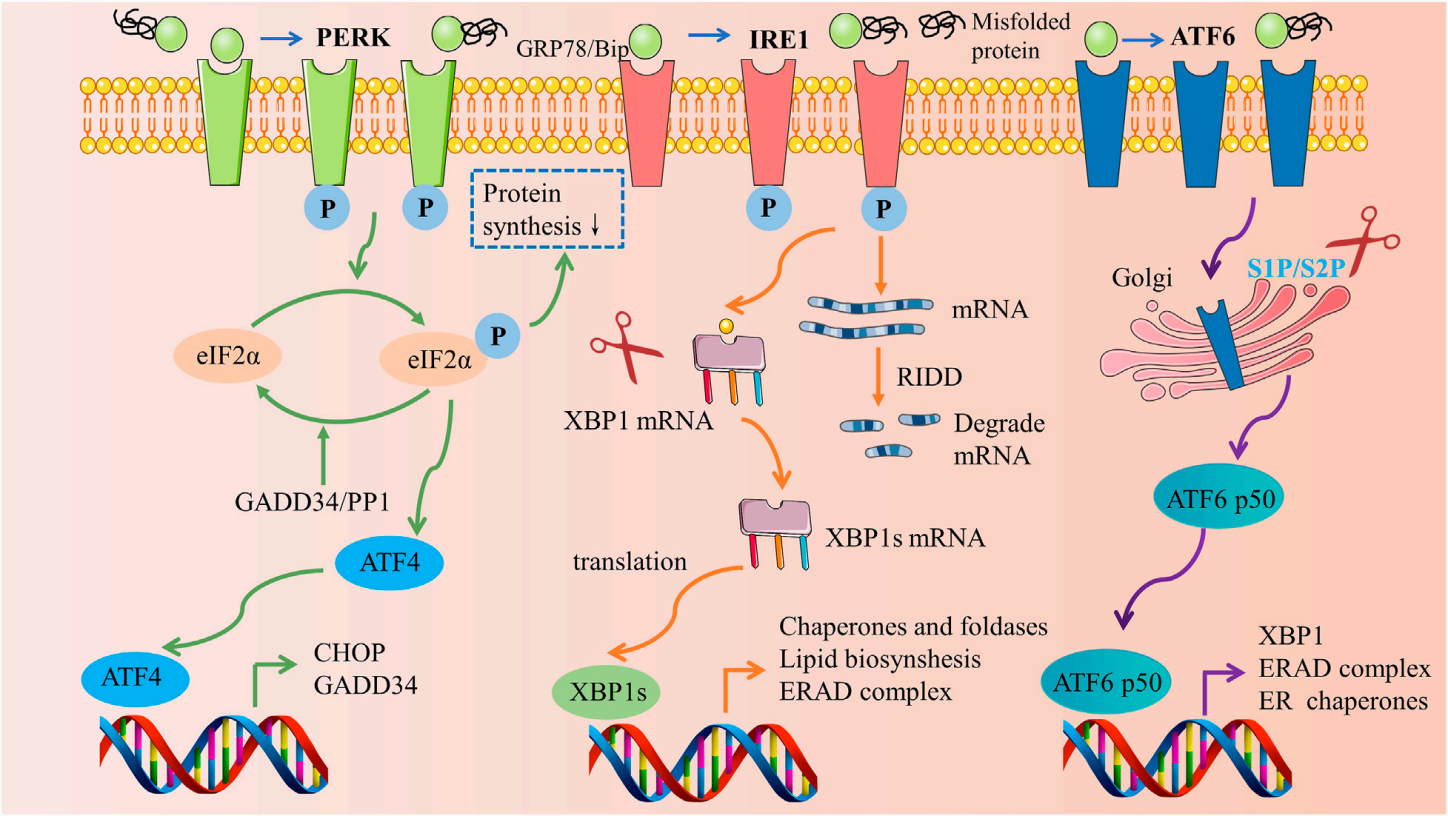
Three signaling pathways of the unfolded protein response. Materials provided by Servier Medical Art (smart.servier.com).

UPR is an adaptive cellular mechanism governed by three ER transmembrane proteins: protein kinase R (PRK)-like ER kinase (PERK), inositol-requiring enzyme 1 (IRE1), and activating transcription factor 6 (ATF6). Under standard physiological conditions, these transmembrane proteins bind to GRP78, thereby maintaining an inactive state. However, when ER stress is induced, an accumulation of unfolded or misfolded proteins results in the binding to GRP78, causing the disassociation of GRP78 from PERK, IRE1, and ATF6. This disassociation instigates the subsequent induction of downstream signaling pathways (Figure 2).

Among these proteins, PERK plays a pivotal role in the modulation of ER stress. Upon liberation from GRP78, oligomerization and autophosphorylation activate PERK (Wang et al., 2018). Once activated, PERK proceeds to phosphorylate the eukaryotic translation initiation factor (eIF2α), thereby mitigating protein synthesis in the ER (Guo et al., 2021). Subsequently, phosphorylated eIF2α selectively enables the translation of activating transcription factor 4 (ATF4) mRNA, concomitantly inhibiting other protein translations, thus fostering the expression of ATF4 (Mukherjee et al., 2020). ATF4 then prompts the generation of two crucial target genes: CHOP and growth arrest and DNA damage-inducible protein (GADD34). While CHOP, a transcription factor, modulates apoptosis-related genes, GADD34 exerts a negative regulation on the PERK pathway by catalyzing the dephosphorylation of eIF2α (Oyadomari and Mori, 2004; Tsaytler et al., 2011).

IRE1 is a type I transmembrane protein located within the ER membrane, exhibiting dual enzymatic activities of serine/threonine protein kinase and endonuclease. Upon the initiation of ER stress, IRE1 undergoes dissociation from GRP78, facilitating a signal transduction across the membrane to the cytoplasmic domain, which subsequently undergoes oligomerization and autophosphorylation. The activated state of IRE1 exhibits endonuclease activity capable of cleaving the mRNA of X-box binding protein 1 (XBP1), thus enabling its translation into the active transcription factor XBP1s. This activated factor translocates into the nucleus, instigating an upregulation in the expression of genes associated with protein folding, thereby mitigating ER stress and reinstating intracellular homeostasis (Yoshida et al., 2001; Bashir et al., 2021). Moreover, IRE1 promotes degradation of numerous ER-targeted mRNAs through the regulated IRE1α-dependent decay (RIDD) cleavage mechanism, impeding their translation and alleviating the protein folding burden on the ER (Hetz et al., 2020).

ATF6, a type II transmembrane protein residing within the ER membrane, possesses a unique mode of action compared to IRE1 and PERK. Upon release, ATF6 is transported to the Golgi apparatus where it is cleaved by site 1 protease (S1P) and site 2 protease (S2P), resulting in the formation of the active form, p50 ATF6 (Haze et al., 1999). Subsequently, p50 ATF6 migrates into the nucleus to bind with the endoplasmic reticulum stress response element (ERSE) located on the promoter, thereby triggering the transcription of XBP1, an ER chaperone protein, and the endoplasmic reticulum-associated degradation (ERAD) complex, all of which contribute to the alleviation of ER stress (Yamamoto et al., 2007).

## 4 The role of ER stress in diabetic nephropathy

### 4.1 Existence of ER stress in diabetic nephropathy

Renal intrinsic cells possess a substantial ER system, thereby establishing the conditions and foundation for the occurrence of ER stress. The renal tissues of patients with progressive, proteinuric DN have reported markedly elevated mRNA levels of HSPA5, HYOU1, and XBP1—principal genes implicated in the UPR (Lindenmeyer et al., 2008). This indicates an activation of ER stress in the kidneys of DN patients. In parallel, Guo et al. (2016) noted the activation of ER transmembrane sensory proteins—ATF6, PERK, and IRE1α—and their downstream targets eIF2α, ATF4, and XBP1 in both *in vivo* and *in vitro* DN models. These findings corroborate that all three branches of the UPR signaling pathway are activated during the course of DN.

A growing body of evidence implicates various factors such as hyperglycemia, proteinuria, free fatty acids, and AGEs in triggering ER stress in renal cells. For instance, Bai et al. (2018) identified that the expression of LINC01619 was downregulated in high glucose-cultured podocytes. This reduction in LINC01619 led to the suppression of FOXO1 expression via acting as a miR-27a “sponge,” thereby initiating ER stress and causing podocyte damage in DN. Sirtuin-1, a nicotinamide adenine dinucleotide-dependent deacetylase, has also been implicated in these processes. A study by Kang et al. (2018) illustrated that sirtuin-1 deacetylated PERK, mitigating the PERK/eIF2α/CHOP ER stress pathway. Furthermore, LncRNA TUG1, downregulated in a high-glucose environment, was found to inhibit sirtuin-1 expression through a sponge-like interaction with miR-29c-3p, which in turn exacerbated ER stress-mediated injury in HK-2 cells (Wang S. et al., 2021). Notably, proteinuria serves not merely as an indicator of glomerular damage but also as a significant risk factor for kidney disease progression. It has been demonstrated that podocytes and renal tubular epithelial cells exposed to high protein loads experience ER stress (Ohse et al., 2006; Gonçalves et al., 2018; Jia et al., 2019). In this context, Jia et al. (2019) discovered that albumin stimulated the expression of miR-4756 in HK-2 cells, which directly suppressed sestrin2 expression by targeting its 3'-untranslated region, consequently inducing epithelial-mesenchymal transition and ER stress in these cells. Another study revealed that simultaneous exposure of HK-2 cells to albumin and high glucose notably enhanced ER stress-related gene expression as compared to exposure to albumin alone (Lindenmeyer et al., 2008).

Palmitic acid, the most abundant free fatty acid in human plasma, has demonstrated profound effects on podocytes, cells particularly sensitive to this fatty acid. Studies by Xu et al. (2015). Highlighted that palmitate stimulates ER Ca^2+^ depletion in mouse podocytes, initiating ER stress. However, this effect was mitigated by silencing the CHOP gene, which attenuated palmitic acid-induced podocyte death (Sieber et al., 2010). In patients with diabetes, AGEs, harmful protein byproducts, are notably elevated and have a propensity to accumulate within all renal structures. Chiang et al. (2016) observed that AGEs trigger ER stress signaling in a time- and dose-dependent fashion. Importantly, the inhibition of ER stress via 4-phenylbutyric acid (4-PBA) successfully reversed AGE-induced apoptosis in mesangial cells. Subsequently, Liu et al. (2015) determined that the ATF4/p16 pathway, regulated by ER stress, contributes to AGE-induced premature senescence of renal tubular epithelial cells. This effect was significantly attenuated by both 4-PBA and ATF4 gene silencing. Moreover; Chen et al. (2008) discovered that AGEs induce GRP78 expression and podocyte apoptosis in a dose- and time-dependent manner, while also triggering a rapid increase in intracellular calcium through the release of ER stores and the influx of extracellular calcium. These effects were substantially diminished following treatment with tauroursodeoxycholic acid; Liang et al. (2016) further demonstrated that salubrinal, a selective inhibitor of eIF2α dephosphorylation, blocked the conversion of human glomerular endothelial cells to mesenchymal cells, instigated by advanced oxidation protein products. Several *in vivo* studies also revealed that ER stress inhibitors substantially reduced proteinuria, ameliorated renal function, and attenuated renal histopathological damage in animal models of DN (Cao A. L. et al., 2016; Fan et al., 2017). Collectively, these findings underscore the presence of ER stress in DN and its critical role in the disease’s onset and progression.

### 4.2 Underlying mechanisms of ER stress involved in the progression of diabetic nephropathy

#### 4.2.1 ER stress and oxidative stress

Oxidative stress is a physiological state characterized by an overproduction of reactive oxygen and reactive nitrogen radicals, surpassing the body’s capacity for oxide removal. This state is typically induced by various harmful stimuli and results in an imbalance between oxidative and antioxidant systems, subsequently leading to tissue or cellular dysfunction (Daenen et al., 2019). Extended periods of ER stress generate a hyperoxic environment within the ER lumen, thereby releasing H_2_O_2_ into the cytoplasm and directly forming cytotoxic intracellular reactive oxygen species (ROS) within the cytoplasm. One empirical human study confirmed that a significant interplay exists between ER stress and oxidative stress, contributing notably to the progression of DN (Victor et al., 2021). In this study, ER oxidase 1α (ERO1α) levels were found to be markedly elevated in the peripheral blood mononuclear cells of DN patients and positively correlated with PERK and p22pHox (Victor et al., 2021). ERO1α, a key intermediary linking ER stress and ROS, also serves as a target of CHOP. A CHOP knockdown mitigated the expression of ERO1α, consequently reducing ROS production (Rao et al., 2015). Moreover, either selective PERK blockade (via GSK2606414) or PERK silencing (shPERK) successfully curtailed the elevated cytoplasmic Ca2+ and intracellular ROS levels (Zhang Y. et al., 2019; Ko et al., 2021). Multiple studies have demonstrated the antioxidant capabilities of ER stress inhibitors. Cao A. et al. (2016) suggested that ursodeoxycholic acid (UDCA) could potentially enhance renal pathology and function by attenuating high glucose-induced oxidative stress. Additionally, Luo et al. (2010) found that 4-PBA effectively inhibited NADPH oxidase activity, reduced malondialdehyde (MDA) levels, and enhanced superoxide dismutase (SOD) activity in DN rats.

#### 4.2.2 ER stress and inflammation

Inflammation serves as a critical mechanism underlying the onset and progression of DN. Recent evidence has implicated ER stress in fostering kidney inflammation under diabetic conditions. Specifically, Zhu et al. (2017) observed that ER stress could incite the expression of CXCL10 and CCL2 via the activation of nuclear factor-κB (NF-κB) and signal transducer and activator of transcription 3 (STAT3) pathways. Notably, these effects were counteracted by PERK knockdown. In the context of the *db/db* mouse model of diabetes, ER stress was found to stimulate the expression of monocyte chemoattractant protein-1 (MCP-1) via the SET7/9-mediated induction of histone methylation. Furthermore, the silencing of the XBP1s gene using siRNA markedly diminished the expression of both SET7/9 and MCP-1 (Chen J. et al., 2014). The NOD-like receptor family pyrin domain containing 3 (NLRP3) inflammasome is known to orchestrate the secretion, maturation, and release of various inflammatory mediators, such as interleukin 18 (IL-18) and IL-1β, that further exacerbate glomerular and tubular damage in DN (Williams et al., 2022). In *β*-cells, ER stress has been found to upregulate the expression of thioredoxin-interacting protein (TXNIP) through the PERK and IRE1 pathways, thereby activating the NLRP3 inflammasome (Oslowski et al., 2012). Consistent with these findings, Yang et al. (2022) demonstrated that chronic ER stress augments the levels of renal pro-inflammatory cytokines, such as TNF-α, MCP-1, IL-1β, and IL-18, by excessively activating the NLRP3 inflammasome. Significantly, the ER stress inhibitor, UDCA, was found to mitigate the bovine serum albumin-induced activation of the NLRP3 inflammasome in renal tubular epithelial cells (Fang et al., 2013).

#### 4.2.3 ER stress and apoptosis

Analogous to the endogenous (mitochondrial) pathway and the exogenous (death receptor) pathway, endoplasmic reticulum stress represents a crucial avenue for apoptosis induction (Hu et al., 2018). Prior research has confirmed that ER stress can steer apoptosis through three pathways: CHOP/GADD153, IRE1/ASK1/JNK, and caspase-12 (Oyadomari and Mori, 2004; Hetz, 2012). Notably, activation of ER-associated apoptotic proteins CHOP, JNK, and caspase-12 in DN rat kidneys was documented as early as 2008 (Liu et al., 2008). CHOP, a pro-apoptotic transcription factor in the ER stress process, resides downstream of PERK. Interventions involving PERK knockdown or CHOP depletion have exhibited protective effects against podocyte apoptosis (Fan et al., 2017; Tian N. et al., 2018). In parallel, HRD1, an E3 ubiquitin ligase, is known to advance eIF2α ubiquitination and degradation. Huang et al. (2017) observed that HRD1 expression was diminished and apoptosis was enhanced in palmitic acid- or high glucose-induced HKC-8 cells. Conversely, overexpression of HRD1 led to decreased p-eIF2α and eIF2α expression, thereby mitigating HKC-8 cell apoptosis. Protein arginine methyltransferase-1 (PRMT1), a key enzyme catalyzing the process of protein arginine methylation, showed marked elevation in DN models. Importantly, PRMT1 knockdown was found to alleviate high glucose- or palmitic acid-induced ER stress and renal intrinsic cell apoptosis through the inactivation of PERK and ATF6 (Park et al., 2017; Chen Y. Y. et al., 2019). A recent investigation demonstrated that KIRA6, a type II IRE1α inhibitor, reversed high-glucose-induced apoptosis in HK-2 cells by attenuating ER stress via the inhibition of IRE1α expression (Xie et al., 2022). In contrast, Xie et al. (2021) reported that podocyte-specific disruption of IRE1α amplified renal cell apoptosis, proteinuria, and renal fibrosis in diabetic mice through the suppression of ADH1 expression. The reasons behind these discrepancies in the role of IRE1α in DN remain elusive, with potential influencers being the differences in cell types and experimental conditions.

#### 4.2.4 ER stress and autophagy

Autophagy represents a highly conserved lysosomal degradation pathway, crucial for maintaining intracellular homeostasis through the degradation of cytoplasmic metabolites and damaged organelles. It has been demonstrated in earlier research that impaired autophagy of renal cells under hyperglycemic conditions plays a pivotal role in the pathogenesis of DN (Koch et al., 2020). More recent investigations have drawn a close connection between ER stress and deficiencies in autophagy. In their study, Cao A. L. et al. (2016) observed that both UDCA and 4-PBA significantly boosted the expression of LC3 A/B II and Beclin-1, thereby mitigating high glucose-induced apoptosis in podocytes. ATF4 serves as a principal regulator of ER stress. Liang et al. (2021) demonstrated that ATF4 knockout resulted in improved urinary albumin levels, renal function, and renal fibrosis in DN mice. Mechanistically, the silencing of the ATF4 gene curtailed p62 and Col-IV protein expression, elevated LC3-II protein expression, and reinstated autophagosomes and autophagic lysosomes in NRK-52E cells cultured under high glucose conditions. In contrast to (Liang et al.) 's findings, Yuan et al. (2021) reported that ATF4 silencing diminished podocyte autophagy triggered by DN mouse serum and heightened podocyte apoptosis. The evidence thus suggests that the influence of ATF4 on the autophagic activity of renal cells during DN pathology may exhibit cell-specific variations. Therefore, future studies could provide further elucidation to reconcile this apparent inconsistency.

#### 4.2.5 ER stress and pyroptosis

**FIGURE 3:**
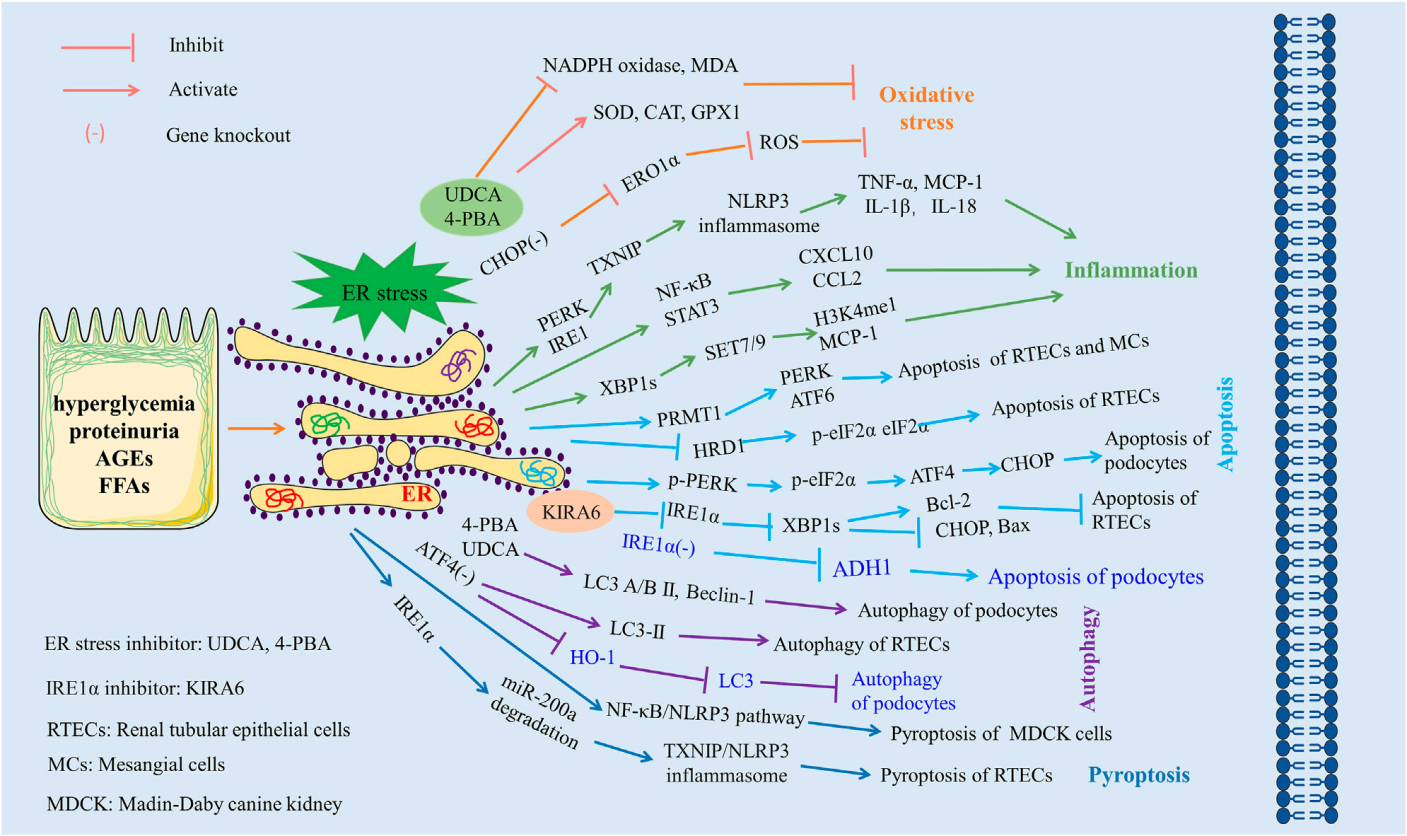
The role of ER stress in diabetic nephropathy. Excessive ER stress exacerbates kidney injury by aggravating oxidative stress, inflammation, apoptosis, and pyroptosis and inhibiting autophagy. Materials provided by Servier Medical Art (smart.servier.com).

Pyroptosis represents a recently identified mode of programmed cell death. It involves the activation of caspases 1, 4, 5, and 11, mediated by inflammasomes, which initiate the cleavage and polymerization of several Gasdermin family members, thereby triggering the formation of cell membrane pores and the subsequent release of inflammatory factors. TXNIP serves as an important molecular link between ER stress and pyroptosis, with TXNIP upregulation being dependent on the activation of PERK and IRE1α (Fu et al., 2021). In DN models, Ke et al. (2020) observed the activation of ER stress, where IRE1α specifically degraded miR-200a. This degradation in turn elevated the TXNIP/NLRP3 inflammasome, which induced pyroptosis in renal tubular epithelial cells and exacerbated renal injury. In line with this, another study corroborated that ER stress-induced pyroptosis via activation of the NF-κB/NLRP3 pathway constitutes a pivotal mechanism of high glucose-induced renal injury (Li et al., 2023). Moreover, the silencing of XBP1 in cadmium-induced HK-2 cells resulted in inhibited NLRP3 inflammasome activation and pyroptosis (Chou et al., 2019). The interrelationships between ER stress and oxidative stress, inflammation, apoptosis, autophagy, and pyroptosis in DN are summarized in Figure 3.

ER stress, a complex biological process, plays a dual role in DN. While the majority of studies to date have reported on the pathogenic implications of ER stress in DN, with excessive ER stress exacerbating pathological processes such as oxidative stress, inflammatory responses, apoptosis, autophagy, and pyroptosis, a few studies intriguingly suggest a potentially protective role for ER stress in DN. Therefore, the question of how to maintain ER stress within a moderate range presents an important direction for future research.

## 5 Modulatory role of Chinese herbal medicine in ER stress in diabetic nephropathy

### 5.1 Chinese herbal formulas

Chinese herbal formulas, comprised of two or more herbs, are a cornerstone of clinical treatments for diabetic kidney disease in China. Recent literature has substantiated the effectiveness of these formulas in improving renal function and reducing proteinuria in patients with diabetic kidney disease (Zhang L. et al., 2019; Liu et al., 2022). Recent investigations have also highlighted a potential link between the amelioration of DN and the regulation of ER stress by certain Chinese herbal formulas (Table 1).

**TABLE 1:** The role of Chinese herbal formula in the regulation of ER stress in DN.

Chinese herbal formula	Experimental models	Effects	Mechanisms	References
Tongluo Yishen formula	Glomerular mesangial cells cultured with high glucose	Reduced apoptosis	JNK, CHOP, and Caspase-12↓, the increase of GRP78↓	Li et al. (2016a); Li et al. (2016b)
		Inhibited ER stress		Li et al. (2016c)
Tongluo Baoshen formula	SD rats injected with 50 mg/kg STZ	Reduced apoptosis	GRP78↓, p-IRE1α↓, p-JNK↓	Zhang et al. (2017a)
		Inhibited ER stress		Zhang et al. (2017b)
Zuogui Jiangtang Yishen decoction	MKR mice treated with high-fat diet and unilateral nephrectomy	Lowered blood glucose urine	GRP78↑, CHOP↓	Tang et al. (2015)
		Reduced urine protein		
		Inhibited ER stress		
Danggui Buxue decoction	SD rats treated with high sugar, high-fat diet and 60mg/kg STZ Glomerular mesangial cells cultured with high glucose	Lowered blood glucose	GRP78↓, PERK↓, eIF2α↓, p-IRE1α↓, ATF6↓, CHOP↓, Caspase-3↓, Caspase-12↓, p-JNK↓	Zhang et al. (2015)
		Reduced urine protein		Zhang et al. (2018)
		Improved renal function		Shuai et al. (2018)
		Reduced apoptosis		Shen et al. (2018)
		Inhibited ER stress		
Modified Shenqi Dihuang decoction	Patients with diabetic kidney disease	Lowered blood glucose	GRP78, ATF6, CHOP, and Caspase-12 ↓	Zhang et al. (2022)
		Alleviated insulin resistance		
		Reduced urine protein		
		Improved lipid profiles		
		Improved renal function		
		Inhibited ER stress		

Glomerular mesangial cell apoptosis is implicated in the worsening of proteinuria and renal function in DN. Prior studies suggest that high glucose can trigger an endogenous pro-apoptotic signaling pathway, leading to mesangial cell apoptosis and subsequent DN progression (Mishra et al., 2005; Lu et al., 2018). *In vitro* studies have shown that the serum containing the Tongluo Yishen formula hindered the surge of GRP78 mRNA and downregulated the expression of c-Jun N-terminal kinase (JNK), CHOP, and caspase-12 mRNA, thereby impeding high glucose-induced apoptosis of glomerular mesangial cells (Li et al., 2016a; Li et al., 2016b; Li et al., 2016c). In the streptozotocin (STZ)-induced diabetic rat model, the Tongluo Baoshen formula treatment led to a considerable reduction in the protein expression of p-IRE1-α, GRP78, and p-JNK in the kidneys, suggesting a renal protective role mediated through ER stress inhibition (Zhang et al., 2017a; Zhang et al., 2017b). Tang et al. (2015) found that the Zuogui Jiangtang Yishen decoction elevated GRP78 expression while lowering CHOP expression in DN mouse podocytes. This implied a possible mechanism of DN retardation through the amelioration of ER stress-mediated podocyte injury. Consistent with this finding, numerous studies have noted that the Danggui Buxue decoction substantially downregulated the expression of ER stress and apoptosis-related proteins in DN rat kidney tissue, including GRP78, PERK, p-IRE1α, ATF6, CHOP, caspase-12, p-JNK, among others (Zhang et al., 2015; Shen et al., 2018; Shuai et al., 2018; Zhang et al., 2018). Further corroborating these observations, Zhang et al. (2022) demonstrated that the modified Shenqi Dihuang decoction not only significantly improved glucolipid metabolism and renal function in DN patients but also exerted a considerable inhibitory effect on serum ER stress and apoptosis markers, such as GRP78, ATF6, CHOP, and caspase-12 mRNA expression. In Supplementary Table S1, we have listed the composition of the above Chinese herbal formulas in detail.

### 5.2 Chinese patent medicine

Chinese patent medicine, an integral component of traditional Chinese medicine, exhibits stable efficacy and offers the advantage of convenient administration. It is extensively utilized in treating diabetic kidney disease to enhance renal function and augment patient clinical outcomes (Sheng et al., 2020; Yu et al., 2022). Certain Chinese patent medicines used in DN treatment, like Chinese herbal formulas, also exert regulatory effects on ER stress (Table 2).

**TABLE 2:** The role of Chinese patent medicine in the regulation of ER stress in DN.

Chinese patent medicine	Experimental models	Effects	Mechanisms	References
Qiwei granule	KK-Ay mice treated with high-fat diet	Lowered blood glucose	GRP78↓, p-IRE1↓, XBP1↓, p-PERK↓, Caspase-12↓	Han and Gao (2019)
		Reduced urine protein		Tian et al. (2019)
	Mouse podocytes cultured with high glucose	Improved renal function		Tian et al. (2018b)
		Reduced apoptosis		
		Inhibited ER stress		
Huangkui capsule	SD rats treated with unilateral nephrectomy, high-fat diet, and 35 mg/kg STZ	Lowered blood glucose	TNF-α↓, IL-6↓, IL-1β↓, IL-2↓,ATF6α↓, p-PERK↓, p-JNK↓	Ge et al. (2016)
		Improved renal function		
		Reduced urine protein		
		Improved lipid profiles		
		Alleviated glomerular injury		
		Suppressed renal inflammation		
		Inhibited ER stress		
Shenyan Kangfu tablet	SD rats treated with 40 mg/kg STZ	Reduced urine protein	GRP78↓, p-JNK↓, CHOP↓	Chang et al. (2017)
		Improved renal function		
		Reduced apoptosis		
		Inhibited ER stress		
Shenshao oral liquid	Wistar rats treated with high-fat diet and 25 mg/kg STZ	Lowered blood glucose	GRP78↓, PERK↓, CHOP↓	Yuan et al. (2017)
		Reduced urine protein		
		Improved renal function		
		Reduced apoptosis		
		Inhibited ER stress		
Shenkang injection	SD rats treated with unilateral nephrectomy and 3 5mg/kg STZ NRK-52E cells cultured with high glucose	Reduced urine protein	E-cadherin↑, α-SMA↓, Vimentin↓, Collagen I↓, Bax↓, Bcl-2↑, Caspase-12↓, Bax/Bcl-2↓, GRP78↓, p-PERK/PERK↓, p-eIF2α/eIF2α↓, ATF4↓, CHOP↓	Wang et al. (2021b)
		Improved renal function		
		Alleviated renal tubular injury		
		Suppressed epithelial-mesenchymal transition		
		Reduced apoptosis		
		Inhibited ER stress		

It has been reported that Qiwei granules can diminish the expression of ER stress-related factors such as GRP78, p-IRE1, XBP1, and p-PERK in the renal tissue of KK-Ay mice. This suggests that Qiwei granules safeguard renal function and alleviate renal pathological damage by inhibiting the activation of IRE1 and PERK pathways (Han and Gao, 2019; Tian et al., 2019). Further *in vitro* studies have revealed that the drug-containing serum of Qiwei granules suppresses the ER stress-mediated caspase-12 apoptotic pathway, thereby reducing podocyte apoptosis (Tian N. X. et al., 2018). Another Chinese patent medicine, Huangkui capsule, an extract from Abelmoschus manihot (L.) Medic, has demonstrated efficacy in reducing proteinuria, improving renal function, and delaying DN progression (Xu et al., 2018). Ge et al. (2016) noted that the Huangkui capsule alleviated renal inflammation and glomerular injury in DN rats in a dose-dependent manner. Mechanistically, Huangkui capsule modulates ER stress by downregulating the expression of ATF6, p-PERK, p-JNK, and JNK proteins. Shenyan Kangfu tablet, commonly used in chronic kidney disease treatment, has been shown to reduce the expression of GRP78, p-JNK, and CHOP in the kidneys of DN rats, suggesting its role in delaying renal function deterioration via inhibiting ER stress-induced apoptosis of renal intrinsic cells (Chang et al., 2017). Yuan et al. (2017) similarly observed that Shenshao oral liquid inhibited the expression of GRP78, PERK, and CHOP proteins in the kidneys of DN rats. Additionally, a separate study found that Shenkang injection effectively inhibited the activity of the PERK-eIF2α-ATF4-CHOP signaling pathway both *in vitro* and *in vivo*. This inhibition serves to mitigate diabetic tubulopathy by restraining renal tubular epithelial-mesenchymal transition and ER stress-induced apoptosis (Wang W. W. et al., 2021). In Supplementary Table S2, we have listed the composition of the above Chinese patent medicine in detail.

### 5.3 Extractive compounds in Chinese herbal medicine

Bioactive compounds derived from Chinese herbal medicine are instrumental in the therapeutic effects of these remedies. They have been identified as significant contributors in the treatment of DN. The present study suggests that the renoprotective effects of these bioactive compounds could be attributed to their ability to modulate ER stress (Table 3).

**TABLE 3:** The role of Extractive compounds of Chinese herbal medicine in the regulation of ER stress in DN.

Category	Extractive compounds of Chinese herbal medicine	Experimental models	Effects	Mechanisms	References
Polyphenols	Curcumins	SD rats treated with 50 mg/kg STZ	Lowered blood glucose	GRP78↓, p-eIF2α/eIF2α↓, ATF4↓, CHOP↓, Caspase-3↓, Bax/Bcl-2↓, JNK↓, Notch2↓, hes1↓	Yu et al. (2020)
			Improved renal function		
		Mouse podocytes induced by angiotensin II	Reduced apoptosis		Deng et al. (2020)
			Inhibited ER stress		
	Epigallocatechin-3-gallate	Mouse podocytes cultured with high glucose	Reduced apoptosis	GRP78↓, p-PERK↓, Caspase-12↓	Xiang et al. (2017)
			Inhibited ER stress		
	Resveratrol	*db/db* diabetic mice NRK-52E cells cultured with high glucose	Reduced urine protein	GRP78↓, CHOP↓, Cleaved Caspase-12↓	Zhang et al. (2020a)
			Reduced apoptosis		
			Inhibited ER stress		
	Chlorogenic acid	SD rats treated with 60 mg/kg STZ	Lowered blood glucose	CAT↑, SOD↑, GSH-Px↑, MDA↓, CHOP↓, ATF6↓, p-PERK/PERK↓, p-eIF2α/eIF2α↓	Zhu et al. (2019)
			Reduced urine protein		
			Improved renal function		
			Attenuated oxidative stress		
			Inhibited ER stress		
Category	Extractive compounds of Chinese herbal medicine	*Experimental models*	Effects	Mechanisms	References
Flavonoids	Chrysin	*db/db* diabetic mice	Reduced urine protein	p-PERK↓, p-eIF2α↓, ATF4↓, CHOP↓, Bax↓, Bcl-2↑, Podocin↑, Nephrin↑, Apaf-1↓	Kang et al. (2017)
		Mouse podocytes cultured with high glucose	Attenuated glomerular and podocyte damage		
			Reduced apoptosis		
			Inhibited ER stress		
	Naringenin	Wistar rats treated with 120 mg/kg nicotinamide and 60 mg/kg STZ	Lowered blood glucose	ROS↓, GSH↑, SOD↑, CAT↑, XBP-1s↓, p-PERK/PERK↓,p-eIF2α/eIF2α↓, ATF4↓, CHOP↓, Bax/Bcl-2↓, Cleaved caspase-3↓	Khan et al. (2022)
			Improved glucose tolerance		
			Mitigated hyperinsulinemia		
		NRK-52E cells cultured with high glucose	Improved renal function		
			Reduced apoptosis		
			Inhibited ER stress		
			Attenuated oxidative stress		
	Total flavones of Abelmoschus manihot	SD rats treated with unilateral nephrectomy and 35 mg/kg STZ HK-2 cells cultured with AGEs	Lowered blood glucose	TNF-α↓, IL-6↓, MCP-1↓,TACE↓, p-iRhom2↓, GRP94↓, XBP1s↓	Liu et al. (2017)
			Reduced urine protein		
			Improved renal function		
			Alleviated glomerulosclerosis fibrosis		
			Inhibited ER stress		
			Suppressed renal inflammation		
Quinones	Emodin	KK-Ay mice treated with high fat diet	Reduced urine protein	GRP78↓, p-PERK↓, p-eIF2α↓, ATF4↓, CHOP↓, Bax↓, Bcl-2↑	Tian et al. (2018a)
			Improved renal function		
		Mouse podocytes cultured with high glucose	Reduced apoptosis		
			Inhibited ER stress		
	Tanshinone IIA	SD rats treated with 60 mg/kg STZ	Lowered blood glucose	GRP78↓, CHOP↓, p-PERK↓, p-eIF2α↓, ATF4 ↓	Xu et al. (2020)
			Improved renal function		
			Inhibited ER stress		
Alkaloids	Berberine	Mouse podocytes induced by palmitic acid	Reduced apoptosis	Cleaved Caspase-3↓, BIP↓, PERK↓, ATF4↓, CHOP↓, ATF6↓, IRE1α↓, Caspase-12↓,ROS↓	Xiang et al. (2021)
			Inhibited ER stress		
			Attenuated oxidative stress		
Terpenoids	Astragaloside IV(a)	SD rats treated with high fat diet and 35 mg/kg STZ	Lowered blood glucose	Bax/Bcl-2↓, Cleaved Caspase-3↓,GRP78↓, p-PERK/PERK↓, ATF4↓, CHOP↓	Ju et al. (2019)
			Reduced urine protein		
			Improved renal function		
			Improved lipid profiles		
			Reduced apoptosis		
			Inhibited ER stress		
	Astragaloside IV(b)	SD rats treated with 65 mg/kg STZ	Reduced urine protein	Bax↓, Bcl-2↑, GRP78↓, p-PERK/PERK↓, p-eIF2α/eIF2α↓, ATF4↓, CHOP↓, TRB3↓	Chen et al. (2014b)
			Reduced apoptosis		
		Mouse podocytes cultured with high glucose	Inhibited ER stress		
	Astragaloside IV(c)	C57BL/6J mice treated with 100 mg/kg STZ	Reduced urine protein	GRP78↓, Cleaved ATF6↓, p-PERK↓, p-eIF2α↓, CHOP↓, p-IRE1α↓, Spliced XBP1↓, TRAF2↓, p-JNK↓, SERCA2↑, Cleaved caspase-12↓, LC3 II↑, Beclin↑, Atg12↑, p62↓	Guo et al. (2017)
			Improved renal function		
		Mouse podocytes cultured with high glucose	Reduced apoptosis		
			Inhibited ER stress		
			Induced autophagy		
	Astragaloside IV(d)	*db/db* diabetic mice	Reduced urine protein	MCP-1↓, TNF-α↓, SERCA2↑, GRP78↓, Cleaved ATF6↓, p-PERK↓, p-eIF2α↓, ATF4↓, CHOP↓, p-IRE1α↓, Spliced XBP1↓, ASK1↓, TRAF2↓, p-JNK↓, Cleaved caspase-12↓, Cleaved caspase-9↓, Cleaved caspase-3↓, Bcl-2↑, Bax↓	Guo et al. (2016)
		Mouse podocytes induced by palmitic acid	Improved renal function		
			Lowered systolic blood pressure		
			Improved glucose tolerance		
			Increased insulin sensitivity		
			Suppressed renal inflammation		
			Reduced apoptosis		
			Inhibited ER stress		
			Restored Ca^2+^ homeostasis		
	Astragaloside IV(e)	SD rats treated with 40 mg/kg STZ	Reduced urine protein	p-PERK/PERK↓, p-JNK/JNK↓, p-eIF2α/eIF2α↓, GRP78↓, ORP150↓,CHOP↓, Cleaved caspase-3↓	Wang et al. (2015)
			Improved renal function		
		Human podocytes induced by tunicamycin	Reduced apoptosis		
			Inhibited ER stress		
Lignans	Arctigenin	*db/db* diabetic mice	Lowered blood glucose	GRP78↓, CHOP↓, Caspase-12↓	Zhang et al. (2019a)
		HK-2 cells cultured with high glucose	Reduced urine protein		
			Reduced apoptosis		
			Inhibited ER stress		
Additional agents	Ginkgo biloba extract EGB761	C57BL/6 mice treated with high fat diet and 50 mg/kg STZ	Lowered blood glucose	a-SMA↓, E-cadherin↑, collagen IV↓, fibronectin↓, GRP78↓, ATF6↓	Han et al. (2021)
			Reduced urine protein		
			Improved renal function		
		HK-2 cells cultured with high glucose	Alleviated renal tubular injury		
			Suppressed epithelial-mesenchymal transition		
			Reduced extracellular matrix accumulation		
			Inhibited ER stress		
	Terpene glycoside component of Moutan Cortex	SD rats treated with high sugar, high fat diet, and 30 mg/kg STZ	Lowered blood glucose	GRP78↓, XBP-1s↓, p-IRE1α↓, IL-6↓, MCP-1↓, ICAM-1↓, p-NF-κB p65↓	Chen et al. (2016)
			Reduced urine protein		
			Improved renal function		
		Rat glomerular mesangial cell line HBZY-1 induced by AGEs	Alleviated glomerular injury		
			Inhibited ER stress		
			Suppressed renal inflammation		
	Total glucosides of peony	Wistar rats treated with 65 mg/kg STZ	Reduced urine protein	GRP78↓, p-PERK↓, p-eIF2α↓, CHOP↓, TXNIP↓	Shao et al. (2017)
			Inhibited ER stress		

#### 5.3.1 Polyphenols

Curcumin, a lipophilic polyphenol derived from *Curcumae Longae Rhizoma*, exhibits antioxidant, anti-inflammatory, anti-apoptotic, renin-angiotensin-aldosterone system regulatory, and anti-fibrotic properties (Yaribeygi et al., 2021). Recent research indicates that curcumin mitigates angiotensin II-induced podocyte damage and apoptosis, partly through ER stress inhibition (Yu et al., 2020). *In vivo* studies confirm that curcumin mediates nephroprotective effects in DN rats by suppressing the activation of the ER stress-mediated apoptotic signaling pathways JNK and Notch2/hes1 (Deng et al., 2020). Epigallocatechin-3-gallate (EGCG), the primary bioactive compound in catechins, has demonstrated significant hypotensive, hypolipidemic, anti-diabetic, and nephroprotective activities in previous studies (Bazyar et al., 2020; Mohan et al., 2020; Zhu et al., 2022). Xiang et al. (2017) reported that EGCG downregulated the protein expression of GRP78, p-PERK, and caspase-12, thereby protecting podocytes against high glucose-induced apoptosis. Resveratrol (RSV), a natural polyphenolic compound, is predominantly found in grapes, peanuts, *Polygoni Cuspidati Rhizoma et Radix*, among other plants. A recent clinical trial suggested that RSV could effectively supplement angiotensin II receptor antagonists, significantly reducing urinary albumin excretion in DN patients (Sattarinezhad et al., 2019). Additional investigations have shown that the mechanism through which RSV improves DN is associated with the suppression of ER stress-induced apoptosis in renal tubular epithelial cells. Specifically, RSV reduces the expression of GRP78, CHOP, and caspase-12 in the DN model (Zhang et al., 2020a). Chlorogenic acid, a widespread dietary polyphenol, exhibits ER stress-inhibiting and antioxidant properties. Zhu et al. (2019) found that chlorogenic acid attenuated the protein expression of renal tissue p-PERK, p-eIF2α, ATF6, and CHOP, augmented the activity of SOD, catalase, and glutathione peroxidase, and reduced MDA levels in a dose-dependent manner. The cumulative evidence suggests that polyphenolic compounds, including curcumin, EGCG, RSV, and chlorogenic acid, could have potential therapeutic applications in DN, largely due to their ability to inhibit ER stress.

#### 5.3.2 Flavonoids

Chrysin, a naturally occurring flavonoid compound, is primarily derived from propolis, *Scutellaria baicalensis*, and *Oroxylum indicum*. Contemporary pharmacological research has demonstrated that chrysin possesses an array of pharmacological properties, including anti-cancer, anti-diabetic, antioxidant, anti-inflammatory, and hepatoprotective characteristics (Naz et al., 2019). Kang et al. (2017) found that chrysin mitigated ER stress via inhibition of the PERK-eIF2α-ATF4-CHOP pathway, thereby improving high glucose-induced podocyte injury and preventing the loss of slit diaphragm proteins. Naringenin, a common dihydroflavonoid, is predominantly found in citrus fruits and Chinese herbs such as Aurantii Fructus Immaturus and Aurantii Fructus. Khan et al. (2022) reported that naringenin augmented the antioxidant capacity of renal cells during hyperglycemic renal toxicity while also demonstrating significant anti-ER stress and anti-apoptotic effects. Naringenin was found to prevent renal tubular epithelial cell apoptosis by diminishing the expression of ER stress-related proteins (p-PERK, p-eIF2α, XBP1s, ATF4, and CHOP) and mitigating disruption to the ER ultrastructure within renal cells. Total flavones of *Abelmoschus manihot* (TFA) represent a total flavonoid component extracted from the flowers of *A. manihot* (L.) Medic. Liu et al. (2017) reported that TFA alleviated renal inflammation and glomerular injury in DN rats by attenuating ER stress and suppressing the activation of iRhom2/TACE signaling.

#### 5.3.3 Quinones

Emodin, a naturally occurring anthraquinone derivative, is prevalent in various Chinese herbal medicines such as *Radix Rhei et Rhizome*, *Polygoni Cuspidati Rhizoma et Radix*, and *Fallopia multiflora* (Thunb.) Harald, among others. This compound exhibits a broad spectrum of pharmacological properties including, but not limited to, anti-inflammatory, antimicrobial, antioxidant, anti-diabetic, anti-fibrotic, immunosuppressive, and hepatoprotective activities (Semwal et al., 2021). Tian N. et al. (2018) reported that emodin treatment ameliorated both renal function and histopathological damage in a DN mouse model. The researchers further established that emodin’s effects were comparable to those of PERK knockdown, with the compound mitigating high glucose-induced podocyte apoptosis by inhibiting the PERK-eIF2α signaling pathway. Tanshinone IIA (Tan IIA), a phenanthraquinone derived from *Salvia miltiorrhiza*, also exhibits a variety of pharmacological activities, such as anti-inflammatory, antioxidant, anti-tumor, and blood circulation-improving effects (Guo et al., 2020). In a STZ-induced DN rat model, Tan IIA showcased hypoglycemic, renal protective, and anti-fibrotic activities. These effects are linked to its ability to inhibit the PERK pathway, thereby mitigating ER stress (Xu et al., 2020).

#### 5.3.4 Alkaloids

Berberine is an isoquinoline alkaloid primarily extracted from the Chinese herbs *Berberis aristata* and *Coptis chinensis*. Extensive research has corroborated its multifaceted biological activities, including lipid-lowering, anti-diabetic, anti-obesity, and anti-tumor effects (Och et al., 2020). A recent clinical investigation confirmed that combining berberine with valsartan treatment significantly outperformed valsartan monotherapy in enhancing renal and vascular endothelial function in patients with diabetic kidney disease (Fang et al., 2017). In another study, berberine was shown to counteract palmitic acid-induced podocyte apoptosis by curbing ER stress and ROS production (Xiang et al., 2021).

#### 5.3.5 Terpenoids

Astragaloside IV (AS-IV), a key bioactive constituent of the traditional Chinese herbal medicine *Astragalus membranaceus*, exhibits an array of pharmacological properties including anti-tumor, anti-diabetic, hepatoprotective, and neuroprotective effects, as evidenced by modern pharmacological studies (Zhang et al., 2020b). Numerous investigations have attributed the nephroprotective capability of AS-IV in DN rats to its inhibition of ER stress (Chen Y. et al., 2014; Wang et al., 2015; Ju et al., 2019). Specifically, AS-IV mitigates apoptosis in diabetic rat renal tubular epithelial cells and podocytes by suppressing the PERK-ATF4-CHOP signaling pathway (Chen Y. et al., 2014; Ju et al., 2019). Recent reports suggest that AS-IV’s capacity to impede podocyte apoptosis is calcium-dependent (Zang et al., 2021). The Sarco/ER Ca^2+^-ATPase (SERCA) plays a pivotal role in maintaining ER Ca^2+^ homeostasis by facilitating the transportation of cytoplasmic Ca^2+^ into the ER. Guo et al. (2017) (Guo et al., 2016); demonstrated that AS-IV’s inhibitory effect on ER stress-mediated podocyte apoptosis correlated with upregulated SERCA2 expression. Notably, the knockdown of SERCA2 markedly dampened the anti-ER stress and anti-apoptotic effects of AS-IV.

#### 5.3.6 Lignans

Arctigenin, a lignan compound derived from *Fructus arctii*, boasts anti-inflammatory, anti-cancer, antioxidant, and immunoregulation properties (Wu et al., 2022). Zhang J. et al. (2019) found that arctigenin significantly reduced blood glucose and urinary protein levels, while mitigating renal pathological damage in db/db mice. At the molecular level, arctigenin inhibited ER stress by downregulating the expression of GRP78, CHOP, and caspase-12 proteins, thereby mitigating high glucose-induced apoptosis in HK-2 cells.

#### 5.3.7 Additional agents

EGB761, a standardized extract of Ginkgo biloba produced by the German Schwabe Company, consists of flavonoids and terpenoids as its primary active components. In a murine model of DN, Han et al. (2021) demonstrated that EGB761 improved renal function and mitigated renal tubular extracellular matrix accumulation and epithelial-mesenchymal transition through ER stress inhibition. The principal ingredient of the Chinese herbal medicine Moutan Cortex is terpene glycoside. Chen et al. (2016) reported considerable nephroprotective and cytoprotective impacts of the terpene glycoside component of Moutan Cortex (MC-TG) in DN models. Mechanistically, MC-TG alleviated ER reticulum stress-associated inflammation by blocking the activation of the IRE1/NF-κB pathway. The total glucosides of paeony (TGP), an active ingredient derived from the root of *Paeonia alba*, possess anti-inflammatory, anti-apoptotic, antioxidant, and immunomodulatory pharmacological properties (Jin and Zhang, 2022). Shao et al. (2017) indicated that TGP significantly diminished urinary protein in diabetic rats, an effect linked to its inhibition of ER stress-related markers and TXNIP expression.

**FIGURE 4:**
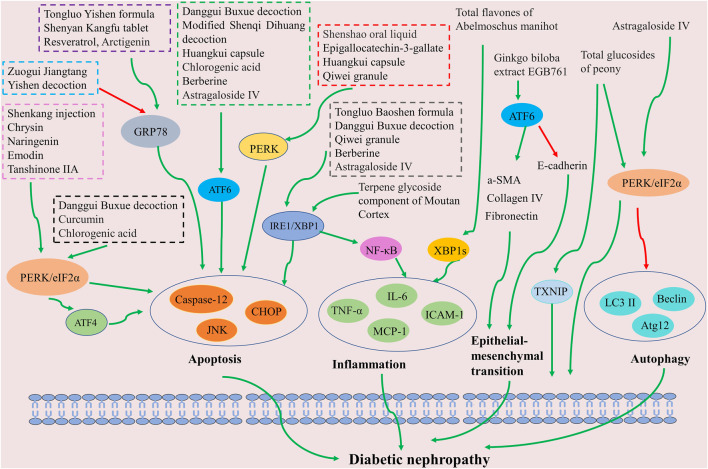
Schematic of Chinese herbal medicine against diabetic nephropathy by inhibiting ER stress. The inhibition of ER stress signaling pathways (including PERK, ATF6, and XBP1) can improve apoptosis, inflammation, autophagy, and epithelial-mesenchymal transition, which benefits diabetic nephropathy treatment.

The aforementioned results imply that Chinese herbal formulas, patent medicines, and extracts—including polyphenols, flavonoids, quinones, alkaloids, terpenoids, among others—may ameliorate DN by inhibiting ER stress (Figure 4). The PERK pathway is the predominant signaling pathway leveraged in Chinese herbal medicine for ER stress inhibition. Attenuating renal intrinsic cell apoptosis is a crucial mechanism of action in providing renal protection after ER stress inhibition by Chinese herbal medicine. In summary, the inhibition of ER stress and associated signaling pathways could potentially represent a significant therapeutic strategy by Chinese herbal medicine to improve DN.

## 6 Conclusion and future directions

As the global incidence of DM escalates, there is a corresponding rise in the number of patients with end-stage renal disease instigated by DM. This trend increases the risk of premature mortality in these patients, amplifying both economic and societal burdens. The etiology of DN is multifaceted, intertwined with a plethora of pathological factors including oxidative stress, inflammation, apoptosis, autophagy, and pyroptosis. The body’s response to detrimental stimuli, embodied by ER stress, exhibits a close association with these pathological processes, collectively promoting the onset and progression of DN.

Empirical clinical data have demonstrated the beneficial effects of certain Chinese herbal medicines, including symptom amelioration, reduction of urinary albumin levels, and renal function preservation in diabetic kidney disease patients. Foundational research corroborates these findings, confirming that ER stress modulation by Chinese herbal medicine can contribute to mitigating renal structural and functional damage, thereby delaying the progression of DN to a certain degree. However, the existing research is not without limitations. The role of Chinese herbal medicine in regulating ER stress has been primarily investigated through the detection of ER stress marker proteins. Whether these herbal medicines directly interact with these proteins or modulate them by influencing upstream signaling pathways warrants further exploration. Moreover, the focus has been predominantly on extracted compounds from Chinese herbal medicine, some of which exhibit disadvantages such as low water solubility, inadequate gastrointestinal absorption, and suboptimal bioavailability. Additionally, the majority of these studies are currently at the preclinical phase, lacking substantial clinical validation. Therefore, future research endeavors should seek to further elucidate the regulatory effects of Chinese herbal medicine on ER stress, overcome challenges related to poor water solubility and low bioavailability of extracted compounds, and initiate high-quality clinical trials. These steps will contribute significantly toward expanding the body of clinical and experimental data supporting the preventive and therapeutic potential of Chinese herbal medicine in the context of DN.
