# Supplementary material for: The role of Chinese herbal medicine in the treatment of diabetic nephropathy by regulating endoplasmic reticulum stress
Source: Front Pharmacol. 2023 Jun 26;14:1174415. doi: 10.3389/fphar.2023.1174415 (PMC10331427; doi:10.3389/fphar.2023.1174415)
Supplement: Supplementary file 1 [file DataSheet1.zip › Supplementary materials (Tables)/Supplementary Table 1.doc]

Supplementary Table 1 The composition of Chinese herbal formulas

| Chinese herbal formulas | Composition |
| --- | --- |
| Tongluo Yishen formula | Prunus persica *(L.)* Batsch, *Achyranthes Bidentata* BI*.*, *Hirudo*, Radix Rehmanniae Recens, *Poria cocos*, *Common Yam Rhizome*, *Alisma orientalis*, *Cornus officinalis Sieb.*, *Herba Epimedii Brevicornus*, *Astragalus membranaceous*, *Winy Rhei Radix et Rhizoma*, and *Radix Puerariae*. |
| Tongluo Baoshen formula | *Astragalus membranaceous*, *Ligusticum chuanxiong* hort, *Cornus Officinalis Sieb.*, *Semen Plantaginis*, *Hirudo*, *Fructus Ligustri Lucidi*, Rheum palmatum L., Salvia miltiorrhiza bunge, and [*Imperatae Rhizoma*](https://old.tcmsp-e.com/tcmspsearch.php?qr=Imperatae Rhizoma&qsr=herb_en_name&token=24db88d311378b2a2cb74d2e1219ed50). |
| Zuogui Jiangtang Yishen decoction | Rehmannia glutinosa (Gaertn.) DC., Salvia miltiorrhiza bunge, *Cornus officinalis Sieb.*, [*Coptidis Rhizoma*](https://old.tcmsp-e.com/tcmspsearch.php?qr=Coptidis Rhizoma&qsr=herb_en_name&token=24db88d311378b2a2cb74d2e1219ed50), *Achyranthes Bidentata* BI*.*, *Astragalus membranaceous*, [*Maydis Seigma*](https://old.tcmsp-e.com/tcmspsearch.php?qr=Maydis Seigma&qsr=herb_en_name&token=24db88d311378b2a2cb74d2e1219ed50), and so on. |
| Danggui Buxue decoction | [*Angelicae Sinensis Radix*](https://old.tcmsp-e.com/tcmspsearch.php?qr=Angelicae Sinensis Radix&qsr=herb_en_name&token=4909a7b4772ca0723233e1ece99ececb) and *Astragalus membranaceous*. |
| Modified Shenqi Dihuang decoction | [*Codonopsis Radix*](https://old.tcmsp-e.com/tcmspsearch.php?qr=Codonopsis Radix&qsr=herb_en_name&token=c6f31f94fa0e173666a2f57bf70eff47), *Astragalus membranaceous*, Rehmannia glutinosa (Gaertn.) DC., *Cornus officinalis Sieb.*, *Poria cocos*, *Common Yam Rhizome*, [*Cortex Moutan*](https://old.tcmsp-e.com/tcmspsearch.php?qr=Cortex Moutan&qsr=herb_en_name&token=c6f31f94fa0e173666a2f57bf70eff47), *Zingiber Officinale Roscoe*, and Jujubae Fructus. |
