# Supplementary material for: The role of Chinese herbal medicine in the treatment of diabetic nephropathy by regulating endoplasmic reticulum stress
Source: Front Pharmacol. 2023 Jun 26;14:1174415. doi: 10.3389/fphar.2023.1174415 (PMC10331427; doi:10.3389/fphar.2023.1174415)
Supplement: Supplementary file 1 [file DataSheet1.zip › Supplementary materials (Tables)/Supplementary Table 2.doc]

Supplementary Table 2 The composition of Chinese patent medicine

| Chinese patent medicine | Composition |
| --- | --- |
| Qiwei granules | *Astragalus membranaceous*, Rheum palmatum L., [*Curcumae Rhizoma*](https://old.tcmsp-e.com/tcmspsearch.php?qr=Curcumae Rhizoma&qsr=herb_en_name&token=957849cb81a20ddd9c93705a59fa3c1b), Radix Rehmanniae Recens, and [*Euonymi Alati Ramulus*](https://old.tcmsp-e.com/tcmspsearch.php?qr=Euonymi Alati Ramulus&qsr=herb_en_name&token=957849cb81a20ddd9c93705a59fa3c1b). |
| Huangkui capsule | *Abelmoschus manihot* (L.) medic. |
| Shenyan Kangfu tablet | [*Panax Ginseng* C.A.Mey.](https://old.tcmsp-e.com/tcmspsearch.php?qr=Panax Ginseng C. A. Mey.&qsr=herb_en_name&token=3b29f5720cf87192e99d64926fc6b7a4), [*Panacis Quinquefolii Radix*](https://old.tcmsp-e.com/tcmspsearch.php?qr=Panacis Quinquefolii Radix&qsr=herb_en_name&token=3b29f5720cf87192e99d64926fc6b7a4), *Radix Rehmanniae Recens*, *Common Yam Rhizome*, [*Eucommiae Cortex*](https://old.tcmsp-e.com/tcmspsearch.php?qr=Eucommiae Cortex&qsr=herb_en_name&token=3b29f5720cf87192e99d64926fc6b7a4), [*Sojae Semen Nigrum*](https://old.tcmsp-e.com/tcmspsearch.php?qr=Sojae Semen Nigrum&qsr=herb_en_name&token=3b29f5720cf87192e99d64926fc6b7a4), [*Hedyotis Diffusae Herba*](https://old.tcmsp-e.com/tcmspsearch.php?qr=Hedyotis Diffusae Herba&qsr=herb_en_name&token=3b29f5720cf87192e99d64926fc6b7a4), [*Smilacis Glabrae Rhixoma*](https://old.tcmsp-e.com/tcmspsearch.php?qr=Smilacis Glabrae Rhixoma&qsr=herb_en_name&token=3b29f5720cf87192e99d64926fc6b7a4), [*Imperatae Rhizoma*](https://old.tcmsp-e.com/tcmspsearch.php?qr=Imperatae Rhizoma&qsr=herb_en_name&token=24db88d311378b2a2cb74d2e1219ed50), *Salvia miltiorrhiza bunge*, [*Leonuri Herba*](https://old.tcmsp-e.com/tcmspsearch.php?qr=Leonuri Herba&qsr=herb_en_name&token=3b29f5720cf87192e99d64926fc6b7a4), [*Platycodon Grandiforus*](https://old.tcmsp-e.com/tcmspsearch.php?qr=Platycodon Grandiforus&qsr=herb_en_name&token=3b29f5720cf87192e99d64926fc6b7a4), and *Alisma orientalis*. |
| Shenshao oral liquid | *Salvia miltiorrhiza bunge*, *Astragalus membranaceous*, [*Radix Paeoniae Rubra*](https://old.tcmsp-e.com/tcmspsearch.php?qr=Radix Paeoniae Rubra&qsr=herb_en_name&token=3b29f5720cf87192e99d64926fc6b7a4), [*Angelicae Sinensis Radix*](https://old.tcmsp-e.com/tcmspsearch.php?qr=Angelicae Sinensis Radix&qsr=herb_en_name&token=4909a7b4772ca0723233e1ece99ececb), *Ligusticum chuanxiong* hort, Prunus persica *(L.)* Batsch, [*Carthami Flos*](https://old.tcmsp-e.com/tcmspsearch.php?qr=Carthami Flos&qsr=herb_en_name&token=3b29f5720cf87192e99d64926fc6b7a4), *Hirudo*, *Pheretima*, and [*Arum Ternatum Thunb.*](https://old.tcmsp-e.com/tcmspsearch.php?qr=Arum Ternatum Thunb.&qsr=herb_en_name&token=3b29f5720cf87192e99d64926fc6b7a4) |
| Shenkang injection | *Astragalus membranaceous*, *Salvia miltiorrhiza bunge*, [*Carthami Flos*](https://old.tcmsp-e.com/tcmspsearch.php?qr=Carthami Flos&qsr=herb_en_name&token=3b29f5720cf87192e99d64926fc6b7a4), and *Rheum palmatum L.* |
